# Risk for Infection with Highly Pathogenic Influenza A Virus (H5N1) in Chickens, Hong Kong, 2002

**DOI:** 10.3201/eid1303.060365

**Published:** 2007-03

**Authors:** Nina Y. Kung, Roger S. Morris, Nigel R. Perkins, Les D. Sims, Trevor M. Ellis, Lucy Bissett, Mary Chow, Ken F. Shortridge, Yi Guan, Malik J.S. Peiris

**Affiliations:** * Massey University, Palmerston North, New Zealand; † University of Hong Kong, Pokfulam, Hong Kong Special Administrative Region, People’s Republic of China; ‡ Department of Agriculture, Fisheries and Conservation, Kowloon, Hong Kong Special Administrative Region, People’s Republic of China; 1 Current affiliation: AgriQuality Ltd, Lower Hutt, New Zealand

**Keywords:** Avian influenza A virus (H5N1), risk factors, case-control study, Hong Kong, research

## Abstract

We used epidemiologic evaluation, molecular epidemiology, and a case-control study to identify possible risk factors for the spread of highly pathogenic avian influenza A virus (subtype H5N1) in chicken farms during the first quarter of 2002 in Hong Kong. Farm profiles, including stock sources, farm management, and biosecurity measures, were collected from 16 case and 46 control chicken farms by using a pretested questionnaire and personal interviews. The risk of influenza A (H5N1) infection was assessed by using adjusted odds ratios based on multivariate logistic regression analysis. Retail marketing of live poultry was implicated as the main source of exposure to infection on chicken farms in Hong Kong during this period. Infection control measures should be reviewed and upgraded as necessary to reduce the spread of influenza A (H5N1) related to live poultry markets, which are commonplace across Asia.

## Abstract 2

Infection was spread to commercial poultry farms through retail marking of live birds.

The spread of highly pathogenic avian influenza (HPAI) type A virus (subtype H5N1) infection in poultry in Asia and beyond poses threats to both human and animal health. Attempts to control outbreaks of this disease in poultry have become a regional and global priority (*1*,*2*). However, little reliable epidemiologic data exist on routes of virus transmission and perpetuation in poultry within affected countries.

Repeated outbreaks of HPAI (H5N1) outbreaks in poultry occurred in farms and live poultry markets within the Hong Kong Special Administrative Region beginning in 1997 (*3*–*6*). The first outbreak was associated with the first recorded transmission of influenza A (H5N1) to humans, with 18 cases and 6 deaths (*7*,*8*). These outbreaks were controlled by slaughtering all poultry in all markets and local farms and stopping all trading of live poultry for 7 weeks. No additional human cases were reported after these interventions, and this particular genotype of influenza A (H5N1) virus has not been detected since (*4*).

No further outbreaks of influenza A (H5N1) in poultry were recorded until 2001, when the virus was detected in live poultry retail markets in Hong Kong. Poultry farms were unaffected. This outbreak led to a slaughter of poultry in live poultry markets in Hong Kong. However, in January 2002, influenza A (H5N1) was again detected in Hong Kong wholesale and retail poultry markets (*3*,*9*). Trace-back from the wholesale poultry market led to detection of the virus on February 1, 2002, on a chicken farm in a densely populated chicken farming area of the New Territories area of Hong Kong. By late March, 17 chicken farms located within 2 km of the index farm, and 4 farms located within 2 to 5 km, were confirmed as infected (*5*,*6*). This outbreak was controlled by a combination of depopulation of infected and contact farms, quarantine and enhanced biosecurity, and vaccination (*10*).

We report the results of an epidemiologic investigation of the 2002 outbreak, including a case-control study to identify risk factors associated with poultry infection in farms. These findings may provide insight into the mechanisms of the spread of HPAI (H5N1) in Asia.

## Materials and Methods

### Study Population

At the time of the 2002 outbreak, 146 commercial chicken farms in Hong Kong were operating, with a combined holding capacity of ≈3 million birds. These farms supplied ≈20% of the poultry consumed within Hong Kong, with the remaining ≈80% imported from farms in the nearby southern provinces of China. Land-based poultry shipments from both Hong Kong and China were usually delivered to 1 wholesale poultry market and then resold to individual live poultry retail markets. Ducks and geese that were reared on poultry farms in China and imported by boat were delivered daily to a geographically separate wholesale market for slaughter and sale as chilled poultry in the live poultry markets.

A cultural preference in Asian countries for freshly killed poultry has resulted in a large volume of sales through live poultry markets, with ≈850 retail poultry market stalls in operation across Hong Kong. Several process changes were introduced in 1998 to reduce the risk of re-introducing the virus into the live market system. Plastic poultry cages were introduced for transporting land-based poultry, and cages that contained poultry from local poultry farms or from China were washed in the wholesale market and then returned to the place of origin. At the wholesale markets, poultry were sorted according to weight and transferred to other washed plastic cages and then transported to the retail poultry markets the next day. Direct sale of poultry from farms to retail markets was discouraged but continued to occur on a limited basis. Details of the live poultry marketing system and compliance requirements imposed by Hong Kong authorities are described separately (N.Y. Kung et al., unpub. data).

After 1997, the Hong Kong government set up a Farm Hygiene section under the Agriculture, Fishery, and Conservation Department for local poultry farm surveillance. This entailed monthly testing for avian influenza and Newcastle disease viruses, testing for serologic evidence of influenza A (H5), and on-farm monitoring of disease and production. Discovery of influenza A (H5N1) in retail poultry markets triggered trace-back, which identified clinically affected farms and led to intensive on-farm investigations that identified more infected farms.

During the 2002 outbreak, clinical disease and influenza A (H5N1) isolations occurred on 22 of the 146 active chicken farms in Hong Kong. For our study, case farms were defined as farms that had high death rates caused by influenza A (H5N1) infection or farms where influenza A (H5N1) was isolated from chickens during the outbreak. Each unaffected farm (n = 124) was assigned a unique identification number, and 46 were selected by using numbers generated with a random number generator in Microsoft (Redmond, WA, USA) Excel for Windows.

### Data Collection

Data were obtained from the 46 unaffected farms and 16 of the 22 case farms. Representatives (farmers or farm managers) from 6 case farms and 3 of the selected control farms were either unavailable or declined to participate in the study. Subsequently, 3 additional unaffected farms were selected by using the random number method to yield the final total of 46 control farms.

Most chickens raised in Hong Kong were sold through 1 wholesale market and distributed from there to retail live poultry markets located throughout Hong Kong. However, some farms also had direct arrangements with retail market stall holders. These chicken farms were concentrated in several areas in the New Territories: Kam Tin, Pak Sha, Ha Tsuen, San Tin, Ngau Tam Mei, and Ta Kwu Ling. The affected farms were clustered in 3 areas: Kam Tin (17), Hung Shui Kiu (1), and Pak Sha (4). Control farms were located in different areas of the New Territories; however, none were in Kam Tin because all the chickens in this region were quarantined and then slaughtered during the January–February 2002 outbreak.

### Survey Methods

We pretested our questionnaire (available from the principal author on request) on 5 chicken farms, then conducted all study interviews during March 2002. The Appendix Table shows the list of potential risk factors surveyed by the questionnaire. Data on geographic location, farm characteristics, stock information, flock health history, farm biosecurity, farm management, and marketing practices were collected by trained interviewers during farm visits. Additional information such as farm area, number of sheds, and incoming day-old chick numbers were obtained from official records held by the Department of Agriculture, Fisheries, and Conservation and used to validate the information collected during on-farm interviews. The questionnaire contained 62 closed and 26 open-ended questions. Of the 88 questions, 77 offered single value and 11 offered multiple value answers.

### Virus Isolation, Subtyping, and Genotyping

Cloacal swab samples were collected from both dead and apparently healthy chickens on farms and tested by using standard procedures for virus isolation (*3*). All influenza virus isolates were subtyped by hemagglutination and neuraminidase inhibition tests by using specific antiserum. Results were confirmed with reverse transcription–PCR specific for influenza A (H5N1). Genetic sequencing and phylogenetic analysis were completed on selected virus isolates (*9*).

### Spatial Analysis

Global positioning system coordinates for all chicken farms in Hong Kong were obtained and entered into a digitized map (Land Information Centre, Survey and Mapping Office, Land Department, Government of Hong Kong) by using a geographic information system program (ArcView 3.1, Environmental Systems Research Institute, Redlands, CA, USA). Coordinates were converted where necessary from latitude and longitude form to map grid on a Hong Kong 80-data format (Survey and Mapping Office, Land Department, Government of Hong Kong) (*11*) to allow for digital mapping and calculation of distances (Figure).

**Figure:**
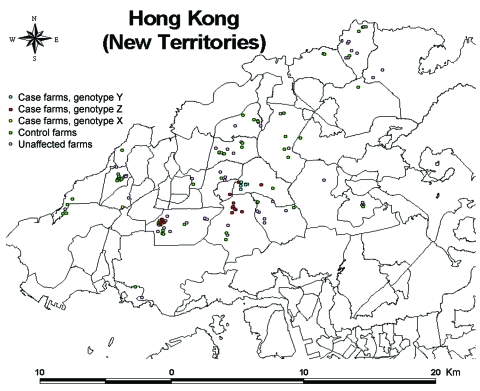
Map of Hong Kong showing the locations of the 22 infected farms (16 case-control study and 6 nonparticipant farms), 46 control farms, and 78 other unaffected farms active during the 2002 outbreak of highly pathogenic avian influenza A virus (subtype H5N1).

### Statistical Procedures

We entered data into a customized database (Microsoft Access 2000) and then transferred it into a statistical package for analysis (SPSS for Windows version 11.0, SPSS Inc., Chicago, IL, USA). We then used descriptive statistics to calculate distributions of all variables by case and control status. We conducted univariate analyses to test for associations between disease status and each explanatory variable by using *t* tests for continuous variables and χ^2^ tests for categorical variables. Where appropriate, we categorized responses before analysis, with categories selected on the basis of the distribution of responses for that variable.

Variables from the univariate analyses with a p value <0.25 were retained for consideration in a multivariate statistical model. Multivariate logistic regression analysis was then used to assess associations between independent variables and the outcome of interest (case or control status), while controlling for other possible risk factors. We constructed the final model by using both forward and backward stepwise procedures. We also used an adapted “best subsets” approach, by which variables of particular interest were forced into the initial equation and the influence of key variables was tested by using the fit of various possible equations. We then assessed model fit by using the Hosmer-Lemeshow goodness-of-fit test (*12*) and the ratio of the deviance to the degrees of freedom. Adjusted odds ratios (ORs) and their 95% confidence intervals were calculated. In all tests, a p value <0.05 was considered significant.

## Results

### Temporal and Spatial Pattern of Genotypes

The Figure shows the locations of the 22 infected farms (16 case-control study and 6 nonparticipant farms), 46 control farms, and 78 other unaffected farms. Three different genotypes of influenza A (H5N1) were identified: 13 case farms were infected with genotype Z, 8 with genotype Y, and 1 with genotype X (Table 1). The spatial pattern showed strong clustering of genotypes Z and Y, with some outliers. The 1 farm infected with genotype X was physically separate from the other 2 clusters. At the time of the outbreak, genotypes Y, Z, and X were isolated from poultry farms, while genotypes Z, B, X_0_, X_1_, X_2_, and X_3_ were detected in live poultry markets. Genotype Y was found only on chicken farms (*9*,*13*).

**Table 1:** Date of identification of avian influenza type A virus (H5N1) infection, farm location, and genotypes for all infected farms, Hong Kong, 2002

Farm ID	Date	District	Genotype
1	1 Feb	Kam Tin	Z
2*	4 Feb	Kam Tin	Z
3	4 Feb	Hung Shui Kiu	X
4	4 Feb	Kam Tin	Y (*4*)
5	4 Feb	Kam Tin	Z
6	6 Feb	Kam Tin	Y
7	6 Feb	Kam Tin	Y
8*	6 Feb	Kam Tin	Y
9	8 Feb	Kam Tin	Z
10	8 Feb	Kam Tin	Z
11	8 Feb	Kam Tin	Z
12	8 Feb	Kam Tin	Z
13	8 Feb	Kam Tin	Z
14	9 Feb	Kam Tin	Y (*4*)
15*	9 Feb	Kam Tin	Y (*4*)
16	15 Feb	Kam Tin	Y
17	16 Feb	Kam Tin	Y
18*	17 Feb	Kam Tin	Z
19	20 Feb	Pak Sha	Z
20	02 Mar	Pak Sha	Z
21*	15 Mar	Pak Sha	Z
22*	18 Mar	Pak Sha	Z

*Infected farms not included in this study.

### Risk Factors for Infection of Farms

#### Univariate Analysis

Statistical comparisons were not done for 9 of the variables from the questionnaire because of either uniformity of response across all farms or excessive missing data. Summary information for farm area, stock numbers, and shed numbers on each farm are presented in Table 2. We performed χ^2^ tests of association on 60 variables in the univariate analysis. Table 3 shows the 19 variables that were associated with a p value <0.25 in the univariate analysis. Affected farms were concentrated in a small number of districts compared with controls, which were more widely distributed across districts (OR 123.0, p<0.01).

**Table 2:** Descriptive analysis of farm area, standing population of chickens, and number of sheds of chicken farms included in survey, Hong Kong, 2002*

	Case (n = 16)					Control (n = 46)					p value
	Mean	Median	Max	Min	SD	Mean	Median	Max	Min	SD	
Farm area (m^2^)	4,008	1,700	18,600	900	4,684	3,275	1,975	28,350	207	4,331	0.86
Chicken count (×1,000)	41.3	27.5	101.4	5.6	33.3	16.2	16.0	51	3.5	10.2	<0.001
Shed no.	7.4	5.5	20	2	5.9	8.0	7.5	19	1	4.4	0.65

*Max, maximum; Min, minimum; SD, standard deviation.

**Table 3:** Results of univariate analysis of risk factors for avian influenza type A virus (H5N1) infection among chicken farms, Hong Kong, 2002*

Variable	Category	Case	Control	OR	95% CI	p value
Farm profile	Pig farm within 500 m	13	29	2.54	0.63–10.21	0.226
	No pig farm within 500 m	3	17	1		
Stock	No. chickens on the farm	NA	NA	NA	NA	<0.001
	Stock density (chicken no./farm area )	NA	NA	NA	NA	0.007
Age group with highest death rate	Most deaths in birds ≥30 d old	5	3	7.40	1.49–36.82	0.017
	Most deaths in birds <30 d old	9	40	1		
Survival rate	≥90% at 1–30 d	15	28	1.54	1.23–1.91	0.003
	<90% at 1–30 d	0	18	1		
	≥90% at 30–60 d	11	42	0.26	0.06–1.22	0.093
	<90% at 30–60 d	4	4	1		
	≥90% at >60 d	13	44	0.20	0.03–1.31	0.103
	<90% at >60 d	3	2	1		
Medication given? (Jan–Feb 2002)	Yes	12	18	4.67	1.30–16.74	0.020
	No	4	28	1		
Were wild birds seen eating in feed troughs?	Yes	10	41	0.20	0.04–0.86	0.036
	No	5	4	1		
Could wild birds gain entry into the shed?	Yes	13	44	0.10	0.01–1.03	0.052
	No	3	1	1		
Does the farm sell chickens directly to a retail market?	Yes	7	3	11.15	2.41–51.56	0.002
	No	9	43	1		
Installed automatic manure scrapers in shed	Yes	11	15	4.55	1.34–15.46	0.018
	No	5	31	1		
Farm owner lives on farm	Yes	7	43	0.05	0.01–0.25	<0.001
	No	9	3	1		
Farm owner owns or partly owns another chicken farm	Yes	3	2	5.08	0.77–33.71	0.103
	No	13	44	1		
Farmer has relatives working in the poultry industry	Yes	4	5	2.73	0.63–11.81	0.219
	No	12	41	1		
Visitors from another chicken farm?	Yes	2	0	0.23	0.15–0.37	0.015
	No	14	46	1		
Visitors from retail markets?	Yes	5	2	10.00	1.71–58.59	0.010
	No	11	44	1		
Visitors went inside sheds?	Yes	7	8	3.94	1.03–15.13	0.040
	No	6	27	1		
District	Within main affected area	15	5	123.00	13.27–1140.46	0.000
	Outside main affected area	1	41	1		

*Variables for which p value is >0.25 have been excluded. OR, odds ratio; CI, confidence interval; NA, not applicable.

Other factors positively associated with case farms: number of chickens on farm; stock density; death rate higher in birds >30 days of age than in younger birds (OR 7.40, p = 0.02); survival rate at 1–30 days of age (OR 1.54, p<0.01); medication use during January–February 2002 (OR 4.67, p = 0.02); whether chickens were sold directly to retail markets (OR 11.15, p<0.01); whether automatic manure scrapers were installed (OR 4.55, p = 0.02); whether persons from retail markets visited during January–February 2002 (OR 10.00, p = 0.01); and whether a visitor went inside the shed during this period (OR 3.94, p = 0.04). Factors that had ORs significantly <1.0 for case farms were reports of wild birds eating in the chicken feed trough (OR 0.20, p = 0.04), farm owner living on farm (OR 0.05, p<0.01), and visitors from another chicken farm during January–February 2002 (OR 0.23, p = 0.02).

#### Multivariate Analysis

Three alternative final models were identified from the model-building procedures, each containing variables that had significant p values (Table 4). Three variables appeared in all models: owner lives off farm, age group with highest death rate at >30 days old, and sale of chickens direct to retail markets. Each model had 1 additional variable, which was different for the 3 models; wild birds in feed trough (protective, model A), number of chickens on farm (model B), and relative working in poultry industry (model C). On the basis of the Hosmer-Lemeshow statistic, model A provided the best fit to the data, while by the adjusted R^2^ statistic, model B had the highest explanatory value. In this model, farms with a nonresident owner were 12.8× more likely to be a case farm; farms that sold chicken directly to retail markets were 30.3× more likely to be a case farm; farms with highest death rate in birds >30 days old were 20.5× more likely to be a case farm; and farms with higher chicken numbers were 1.1× more likely to be a case farm.

**Table 4:** Comparison of different multivariate models of risk factors for avian influenza type A virus (H5N1) infection among chicken farms, Hong Kong, 2002**

Variable	Category	OR (95% CI)		
		Model A	Model B	Model C
1	Owner lives off farm	37.04 (3.18–431.63)	12.64 (1.18–135.35)	45.84 (3.65–575.69)
2	Sell to retail markets	20.11 (1.47–274.98)	30.26 (2.26–405.09)	28.39 (2.30–350.40)
3	Highest death rate >30 d	17.37 (1.03–292.01)	20.51 (1.51–277.96)	24.28 (1.62–364.87)
4	Wild birds in feed trough	0.07 (0.01–0.85)		
5	Chicken count		1.07 (1.01–1.12)	
6	Relative in poultry industry			19.41 (1.46–257.74)
Cox and Snell R^2^		0.42	0.46	0.43
Nagelkerke R^2^		0.63	0.68	0.65
Significance of Hosmer and Lemeshow test		0.91	0.67	0.82
Degrees of freedom		3	8	3

*OR, odds ratio; CI, confidence interval.

Residual components from all 3 models showed 1 farm (Farm ID 19) with a large standardized residual. This was a farm in Pak Sha area where influenza A (H5N1) was isolated on February 20, 2002, but the model predicted it would be a control farm. Farm 19 imported day-old chicks from China during mid-February and sold some chicks 10 days later to another nearby farm (case farm, Farm ID 20). Infection may have entered this farm directly with imported birds; therefore, it did not share risk factors with the other case farms.

## Discussion

We describe the use of a combination of descriptive and analytic epidemiologic techniques to investigate possible risk factors associated with the 2002 influenza A (H5N1) outbreak in Hong Kong. The small sample size limited the number of risk factors we could combine in a multivariable model and prevented consideration of interaction terms. Models containing ≥4 variables and models containing interaction terms either did not converge or showed evidence of multicollinearity. Inspection of counts for combinations of explanatory variables indicated that these occurrences were likely to be the result of zero counts. Because the study included only 16 case farms and related directly to transmission processes that exposed these specific farms, our inferences apply only to the specific circumstances of this outbreak, and caution should be used in applying these findings to other situations.

Comparison of the clusters of case farms with the spatial distribution of randomly selected control farms indicated strongly that locally operating contagious risk factors had a strong influence on which farms become infected. That is, either the virus was spread between farms or nearby farms were exposed to a common source of infection.

Many of the commercial chicken farms in Hong Kong operate as small, family-run businesses and are located in close proximity to each other. Some larger scale commercial chicken farms (concentrated in the Kam Tin area) operate with modern facilities such as automatic manure scrapers, drink dispensers, and feeders. On all of these farms except 1, chickens are raised in 3- to 4-tier cages located in open-sided sheds with fan-assisted ventilation. These farms also use a “continuous flow of stock” operation, which means they contain different age groups of chickens at any 1 time.

Only 1 chicken farm in Hong Kong at the time of the 2002 outbreak operated on an “all-in, all-out” basis, with ≈9,000 chickens at same age group raised in 2 levels of open (not individual), net wire–fenced area. Consequently, this farm had less contact with markets and enhanced biosecurity compared with other case farms. A notable point is that this farm was the only case farm located outside the main affected district and the only farm where the X genotype was isolated during the 2002 outbreak. Joint interpretation of the epidemiologic investigation findings and gene sequence results shows that the disease apparently entered a small number of chicken farms as a single transmission event and then either was controlled on that farm (the geographically isolated farm affected by genotype X) or spread laterally to farms that shared local exposure factors (farms clustered in certain areas affected by genotypes Y and Z).

Visits to a farm by ≥1 persons from retail markets was a strong risk factor for infection. This supports the hypothesis that infection began in the retail markets, where locally produced and imported poultry were mixed and kept for several days (*14*). A US study showed that avian influenza virus (subtype H5N2) amplified in the retail poultry market setting (*15*). Research in Hong Kong has shown that that “rest days,” when markets are emptied of all poultry and cleaned, can interrupt virus perpetuation (*16*,*17*). Therefore, influenza A (H5N1) introduced into poultry markets in 2002 likely was amplified within them and transmitted back to a few index farms, initiating each genotype-specific outbreak. Each genotype then spread to other farms (Y, Z) or remained limited to the index farm (X), depending on the proximity and operation design of the farm. The virus may also have been carried among farms by retail poultry market personnel who visited multiple farms.

Factors that require particular attention in risk management include movement of humans (e.g., buyers, bird catchers) and inanimate objects (e.g., cages, trucks) between retail markets and farms, or among multiple farms, because these movements may carry virus in ways that expose birds to an infectious dose. Airborne spread from affected birds, either while infection was spreading within a flock or during slaughter of a flock, may explain a small number of cases (especially those associated with the Y genotype), but most secondary cases appeared to be due to transfer of virus between farms in ways that could be prevented with enhanced biosecurity.

In addition, influenza A (H5N1) has been isolated from terrestrial birds (*13*,*18*,*19*), which raised the concern that local resident wild birds could introduce virus into a flock. However, although the presence of wild birds in the vicinity of the chicken farms was considered a possible risk factor for introducing avian influenza, it was not significant in this analysis. In fact, wild birds being observed in feed troughs was a protective factor for infection cases in both univariate and multivariate analyses. This information should be interpreted with caution, however, because the operators of case farms underwent questioning by government field officers after the farm was identified as infected and thus may have been more aware of the possibility of transmission of avian influenza from wild birds. This may have increased the frequency with which case farms reported of the presence of wild birds in feed troughs in case farms compared with control farms.

The death rate in chickens >30 days old was higher on case farms than on control farms, which is to be expected because avian influenza kills chickens of all ages and will increase the death rate in older age groups. In addition, chickens in this age group were more likely to be visited by the stock agents, catchers, or farmers before being sent to the markets. Notably, all 3 models showed that the owner living off the farm was a significant risk factor. These farms may have outside visitors, or the owners may be more likely to employ nonfamily workers, and this increased activity increases the likelihood that the virus will be brought onto the farm. Owners who live on the farm may also be more attentive to implementation of protective measures.

The evidence from this study points toward influenza A (H5N1) moving from retail markets to farms for each of the genotype-specific outbreaks. Genotype Y was not isolated from retail poultry market samples at the time of the outbreaks, likely because of the relatively small numbers of live poultry markets that were under routine virologic surveillance at that time. However, we cannot rule out the possibility of an alternative route of introduction of this genotype into the farms, e.g., through wild birds or smuggled poultry.

Enhancement of farm biosecurity would be a useful measure to reduce entry of virus onto farms and interfarm spread. Good farm management and strict biosecurity measures are beneficial actions available to prevent entry of infection to farms and transmission between sheds within farms (e.g., only allowing authorized persons to enter the farm, providing a change of clothes and footwear for all visitors, requiring a stand-down period for anyone who had been in retail poultry markets, ensuring strict control of equipment and transport vehicles entering farms). The role of live poultry markets in the amplification and dissemination of influenza viruses is likely to be related to the maintenance of HPAI (H5N1) across Asia, where such live poultry markets serve the demand for the consumption of freshly killed poultry. One way of reducing the risks associated with live poultry marketing is to reduce the levels of virus circulating in these markets, which has been achieved in Hong Kong through a combination of compulsory vaccination and strict biosecurity measures on poultry farms.

## Supplementary Material

Appendix Table Items in case-control study questionnaire to investigate avian influenza type A virus (H5N1) virus infection, Hong Kong, 2002
