# Supplementary material for: Risk for Infection with Highly Pathogenic Influenza A Virus (H5N1) in Chickens, Hong Kong, 2002
Source: Emerg Infect Dis. 2007 Mar;13(3):412–8. doi: 10.3201/eid1303.060365 (PMC2725907; doi:10.3201/eid1303.060365)
Supplement: Appendix Table — Items in case-control study questionnaire to investigate avian influenza type A virus (H5N1) virus infection, Hong Kong, 2002 [file 06-0365_appT-s1.pdf]

Appendix Table. Items in case-control study questionnaire to investigate avian influenza type A virus (H5N1) virus infection, Hong Kong, 2002

Items	Factors
Farm profiles	
Physical description	Location, area, topography, truck accessibility
Other animals	Pig farm, other species (include pet birds, wild birds, etc)
Pond, stream	Inside, outside, wild birds
Fence	Full, incomplete
Water source	Town supply, well, mountain water, tank cover
Stock	
Chicken and others	Number
Day-old chicks	Source, numbers, unload area (outside, inside farm)
Local farm chickens	Buy in, sell to
Vaccination history	Age administered, types, manufacturers and suppliers of vaccines
Flock health history	Respiratory, digestive, neurologic symptoms, demeanor
Survival rates	Age group, time period before outbreak
Medication to chicken	Types, suppliers, date of administration
Feed source	Types, suppliers, date of delivery, mode of transportation
Feed on farm	Sell to other farms, wild birds in feed store, feed trough
Water system	Drip, cup, trough
Sell to market	Date, number, catcher, retail/wholesale
Cages	Source (retail, wholesale), cleanliness
Pick-up truck	Enters farm
Sheds	
Shed	Number, ventilation, floor cover, wild birds access
Free-range	Age groups, types and sources of bedding materials
Stocking density	Cage size, number of chickens per cage
Cage cleaning post-sale	Method, detergent used, cage empty time
Manure scraper	Installation, frequency
Farm management	
Owner	Live on farm, own other farm, wholesale or retail stall, relatives in poultry industry
Farm workers	Number, origin
Visit outside farm	Mainland, other local farm
Visitors	Number, occupation (farmer, wholesaler, retailer, feed company), enter shed
Biosecurity	Detergent at entrance, visitors wear protective cloth while entering the shed
Transportation	Chicken, feed
Manure disposal	Frequency of collection, truck enters the farm
Waste water disposal	Soak away tank or other, tank cover
Dead chicken disposal	Dead stock collection point, waste pit on farm
